# A “Graft to” Electrospun Zwitterionic Bilayer Membrane for the Separation of Hydraulic Fracturing-Produced Water via Membrane Distillation

**DOI:** 10.3390/membranes10120402

**Published:** 2020-12-07

**Authors:** Yu-Hsuan Chiao, Micah Belle Marie Yap Ang, Yu-Xi Huang, Sandrina Svetlana DePaz, Yung Chang, Jorge Almodovar, S. Ranil Wickramasinghe

**Affiliations:** 1 Department of Chemical Engineering, University of Arkansas, Fayetteville, AR 72701, USA; ychiao@uark.edu (Y.-H.C.); ssdepaz@uark.edu (S.S.D.); 2 R&D Center for Membrane Technology, Chung Yuan Christian University, Taoyuan City 320, Taiwan; mbmyang@gmail.com (M.B.M.Y.A.); changyung0307@gmail.com (Y.C.); 3 School of Environmental Science and Engineering, Sun Yat-sen University, Guangzhou 510275, China; huangyx253@mail.sysu.edu.cn

**Keywords:** antifouling, electrospinning, flux, fouling, hydrophilic, membrane distillation, omniphobic, produced water, water treatment

## Abstract

Simultaneous fouling and pore wetting of the membrane during membrane distillation (MD) is a major concern. In this work, an electrospun bilayer membrane for enhancing fouling and wetting resistance has been developed for treating hydraulic fracture-produced water (PW) by MD. These PWs can contain over 200,000 ppm total dissolved solids, organic compounds and surfactants. The membrane consists of an omniphobic surface that faces the permeate stream and a hydrophilic surface that faces the feed stream. The omniphobic surface was decorated by growing nanoparticles, followed by silanization to lower the surface energy. An epoxied zwitterionic polymer was grafted onto the membrane surface that faces the feed stream to form a tight antifouling hydration layer. The membrane was challenged with an aqueous NaCl solution containing sodium dodecyl sulfate (SDS), an ampholyte and crude oil. In the presence of SDS and crude oil, the membrane was stable and displayed salt rejection (>99.9%). Further, the decrease was much less than the base polyvinylidene difluoride (PVDF) electrospun membrane. The membranes were also challenged with actual PW. Our results highlight the importance of tuning the properties of the membrane surface that faces the feed and permeate streams in order to maximize membrane stability, flux and salt rejection.

## 1. Introduction

Today, the United States is the largest crude oil exporter in the world with a crude oil production of 3.6 million barrels per day [1]. The growth in crude oil production is due in large part to the rapid development of hydraulic fracturing technology. However, hydraulic fracturing requires the use of large amounts of water, around 2000–4600 m^3^ per well [2]. This hydraulic fracturing flow back and produced water (PW) is highly impaired. Typically, it is deep well injected, used to stimulate new wells, or discharged. However, all three disposal options require various degrees of water treatment. Treating the recovered water for beneficial uses is the most environmentally sustainable solution. Membrane-based treatment technologies are appealing as membrane modules are easily scalable, lightweight and have a small footprint. Thus, the unit could be moved to different wellheads as required. However, the water is highly impaired, often containing very high total dissolved solids (TDS) at more than 100,000 ppm. Further, the presence of dissolved polar and non-polar organic compounds as well as surfactants means that membrane fouling is a major concern [3]. 

Pressure-driven and osmotically driven membrane processes, such as reverse osmosis (RO), nanofiltration (NF), ultrafiltration (UF), microfiltration (MF) and forward osmosis (FO), have been used to purify different types of wastewater. However, in the case of the highly impaired PW investigated here, these treatment technologies are generally not practical as significant pretreatment of the feed would be required. A promising thermal-based membrane separation technology–membrane distillation (MD)–can help reduce the worldwide water–energy stress in a sustainable manner [4]. Here, we focus on MD. The driving force for MD is the vapor pressure difference between the feed and permeate sides. Unlike RO, the driving force is less sensitive to high TDS [2,3,4,5,6,7,8].

We have investigated the use of direct contact membrane distillation (DCMD) where the feed and permeate streams are in direct contact with the membrane. This configuration has been frequently used because of its simplicity and lower operational costs compared to other configurations [9]. Hydrophobic membranes are used as the aim is to allow water vapor to pass through the membrane pores and condense on the permeate side. Dissolved, non-volatile species will be concentrated in the retentate. Volatile species will also pass into the permeate. However, organic compounds and surfactants will adsorb onto the surface of typical commercially available hydrophobic membranes such as polytetrafluoroethylene (PTFE), polyvinylidene difluoride (PVDF), and polydimethylsiloxane (PDMS).

Designing membranes that suppress fouling by polar and non-polar organic species is challenging. Recent efforts have focused on developing omniphobic surfaces [10]. However, omniphobic surfaces are fouled by low surface energy compounds such as surfactants. Thus, simply lowering the surface energy of the surface is insufficient. These omniphobic surfaces should contain a re-entrant structure which provides a kinetic barrier to adsorption of low surface energy compounds [11].

Electrospun membranes containing nanofibers with a re-entrant structure could provide an ideal base membrane which could be surface modified in order to impart omniphobic surface properties [12,13,14]. For example, nanoparticles have been attached to the surface of electrospun PVDF membranes in order to create a hydrophobic lotus-like structure through electrostatic [15] or chemical bond interactions [4,16,17]. An omniphobic surface was created by further coating the surface with a fluorine-containing material.

Several studies have been reported the use of hydrogels [18], ionic liquids [5], chitosan [15], and polyethylene glycol (PEG) to tune the surface hydrophilicity and hydrophobicity of the MD membrane [19]. Among the different methods explored, tuning the surface using zwitterions is the most attractive method which could also enhance the antifouling properties of the membrane due to the presence of oppositely charged moieties on the same segment. Moreover, zwitterion augmented membranes could prevent both positive and negative charged foulants from adsorbing onto the membrane surface [3,20,21].

Several methods have been used to modify membranes for MD applications such as physical coating and free-radical polymerization. These methods suffer from disadvantages such as instability of the coating for long-term operation and loss of the antiwetting property due to polymerization inside the pores. Ideally, only the outer membrane surface should be modified. Compared to other methods, the “grafting to” method where a performed polymer is grafted to the membrane surface appears to be a promising method that could be commercialized [22,23]. Due to diffusion limitations and steric hindrance effects, grafting is suppressed on the inside pore surface.

In this study, we have developed bilayer electrospun MD membranes. The membrane surface that faces the feed consists of an oleophobic surface to prevent foulant adsorption. The membrane surface that faces the permeate steam consists of an omniphobic surface that suppresses scale formation and low-surface tension compounds from wicking and fouling the membrane. The membrane surface the contacts the feed stream was modified with a zwitterionic co-polymer poly (glycidyl methacrylate-sulfobetaine methacrylate) (GS) grafted to the membrane surface using self-assembly between the hydroxy groups on the surface generated by alkaline treatment and the anchor segment epoxy group on GS. The membrane surface that contacts the permeate stream was modified by growing silica nanoparticles through electrochemical interactions followed by coating with a fluorine monomer, 1H,1H,2H,2H-perfluorodecyltriethoxysilane (FAS), as a support layer for maintaining the membrane’s antiwetting property. In order to determine the practical viability of these novel membranes, membrane performance was investigated using hydraulic fracturing-produced water obtained from a commercial facility.

## 2. Material and Methods

### 2.1. Materials

All reagents used in this study were of ACS reagent grade without any purification unless otherwise specified. Pelleted poly(vinylidene fluoride-co-hexafluoropropylene) (PVDF-HFP), sodium chloride (NaCl), and silica nanoparticles (Ludox HS-40) were purchased from Sigma-Aldrich (St. Louis, MO, USA). Dimethylformamide (DMF), acetone, ethanol, hexane, cetyltrimethylammonium bromide (CTAB), sodium hydroxide (NaOH), glycidyl methacrylate (GMA), azobisisobutyronitrile (AIBN), and sulfobetaine methacrylate (SBMA) were purchased from VWR (Atlanta, GA, USA). The 1H,1H,2H,2H-perfluorodecyltriethoxysilane monomer (FAS, Matrix Scientific, Columbia, SC, USA) was used to decorate the silica nanoparticle on the PVDF electrospun membrane. Mineral oil was purchased from Equate (Rogers, AR, USA) and was used to characterize surface hydrophobicity.

### 2.2. Synthesis of the Zwitterion Copolymers

Similar to our previous work [24], poly(glycidyl methacrylate-sulfobetaine methacrylate) (pGS, M.W. 62.52 kD) was used to modify the electrospun PVDF membrane through a “grafting to” approach. pGS was synthesized through free radical polymerization of SBMA and GMA. Briefly, GMA and SBMA were dissolved separately in a 25 wt% aqueous methanol solution. These solutions were placed into a round-bottom flask and mixed for 10 min. The mole ratio of GMA to SBMA was kept at 2:8. Afterwards, 0.15 g AIBN was gently added to the flask, and the solution was stirred for 15 min. After obtaining a homogeneous solution, the reaction was continued for 6 h at 60 °C in a nitrogen environment. The reaction solution was cooled in an ice bath. The white precipitate was purified twice using 1 L of methanol. The pGS copolymer was obtained through lyophilization.

### 2.3. Fabrication and Modification of MD Membrane

#### 2.3.1. Electrospun Membrane Fabrication

The membranes were fabricated using a homemade laboratory scale electrospinning apparatus consisting of a syringe pump (NE-1000, New Era pump system Inc., Farmingdale, NY, USA), power supply (Gamma High Voltage Research Inc., Ormond, FL, USA), collector drum (Falco Co., Ltd., New Taipei City, Taiwan), and horizon reciprocating stage (Membrane Science Inc., Hsinchu, Taiwan) in a non-conducting plastic box. The temperature and humidity of the environment were controlled at 23 °C and 55%, respectively.

**Figure 1:**
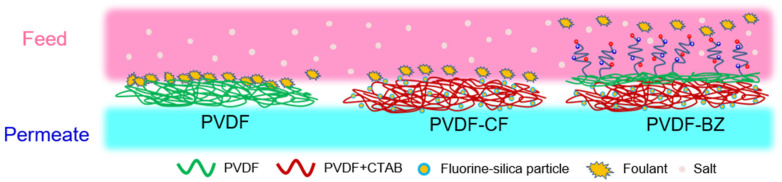
Schematic representation of the polyvinylidene difluoride (PVDF), PVDF-CF, and PVDF-BZ membrane structure.

Figure 1 illustrates the electrospun membrane structures we have developed consisting of the base electrospun PVDF membrane (labelled PVDF), base PVDF membrane with and omniphobic surface (labelled PVDF-CF), and bilayer PVDF membrane (PVDF-BZ). To prepare the base PVDF membrane, a 16 wt% PVDF-HFP solution in 7:3 DMF/acetone was prepared and stirred for 24 h at 35 °C. The solution was degassed overnight at room temperature. Next, the solution was placed in a syringe with a stainless-steel needle. The syringe was placed in the syringe pump of the electrospinning apparatus. The flow rate and voltage were maintained at 1 mL min^−1^ and 14 kV, respectively. The rotation speed of the collector drum was adjusted to 140 rpm, and the distance between the needle and the collector drum was kept at 15 cm. After spinning for 6 h, the membrane was removed from the aluminium foil that covered the collector drum and was air dried in a fume hood to evaporate the residual solvent. The omniphobic PVDF-CF membranes were fabricated similarly to the base PVDF membranes using a two-step process. Initially, 50 mg of CTAB, a positively charged species, was added in the PVDF doping solution. Due to the high charge density of the polymer solution, 23 kV was applied during electrospinning. Next, a base PVDF layer was spun on top of the PVDV-CF structure. The spinning time for the PVDF-CF layer was 5.5 h, followed by 0.5 h for the base PVDF layer. The bilayer was modified as described in Section 2.3.2.

#### 2.3.2. Membrane Modification

**Figure 2:**
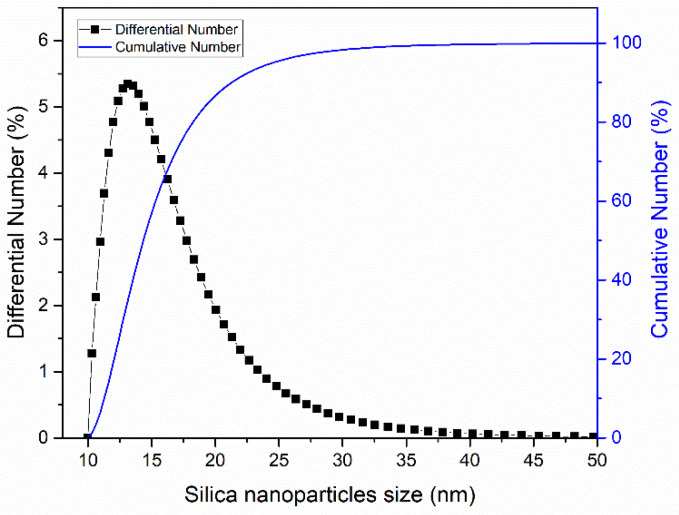
Distribution of silica nanoparticle size. This result was obtained using dynamic light scattering (DLS; Delsa™Nano, Beckman Coulter, Inc, Brea, CA, USA).

Silica particles were grown on the PVDF-CF layer by electrostatic interaction using dip-coating. The PVDF-CF membrane was immersed in 0.2 wt% silica particles in acetate buffer solution (pH~4) for 1 h. The size of the silica nanoparticles was about 15.8 ± 4.8 nm (Figure 2). The membranes were gently rinsed using deionized (DI) water then air dried overnight. Next, the membrane was transferred to a 1 wt% FAS/hexane solution for 24 h.

Fabrication of the bilayer PVDF-BZ membrane was by alkaline treatment to generate hydroxyl surface functionality for zwitterionic grafting [5]. The PVDF layer was placed in a 7.5 M sodium hydroxide (NaOH) solution for 30 min. Due to the hydrophobicity of the PVDF layer, the membrane floated on the solution during alkaline treatment. Next, the membrane was gently rinsed with DI water to remove any residual solution. Modification of the membrane with the zwitterionic polymer was performed by the “grafting to” method. Briefly, 100 ppm GS solution at 65 °C was prepared and the PVDF layer of the PVDF-BZ membrane was placed in the solution, enabling grafting of the GS polymer by reaction with the hydroxyl groups present.

### 2.4. Membrane Characterization

All membranes were rinsed with DI water and then dried before characterization. All membrane surfaces were characterized using attenuated total reflectance Fourier transform infrared spectroscopy (ATR–FTIR; Perkin Elmer Spectrum 100 FT–IR Spectrometer, Waltham, MA, USA). Water, ethanol and underwater oil contact angles were determined (model OCA15EC, Future Digital Scientific, Garden City, NY, USA). These contact angles provided information on surface hydrophobicity, omniphobicity, and surface energy. Membrane morphology was determined using a scanning electron microscope (SEM; FESEM S-4800, Hitachi Co., Tokyo, Japan). Surface charge was determined by measuring surface streaming potential (SurPASS Electrokinetic Analyzer, Anton Paar, Ashland, VA, USA). The oil droplet sizes in the aqueous feed streams were determined by dynamic light scattering (Beckman Coulter, Brea, CA, USA).

### 2.5. Membrane Performance

Membrane performance was determined using a custom laboratory-scale DCMD apparatus. The apparatus consisted of a membrane cell, two gear pumps, heating and cooling systems, a conductivity meter and a digital balance. The active membrane area in the PTFE membrane cell (Membrane Science Inc., Hsinchu, Taiwan) was 2 cm × 4 cm (0.0008 m^2^). Permeate flux and salt rejection were determined using a synthetic feed stream consisting of 3.5 wt% (35,000 ppm) NaCl. The feed tank, 0.8 L in volume, was maintained at 60 °C. The permeate tank, 1 L in volume, contained deionized (DI) water at 20 °C. Feed and permeate flow rates were maintained at 31.2 cm s^−1^ (Reynolds number (Re) is ~1435) and 12.7 cm s^−1^ (Re: ~584) using a gear pump (WT3000, Longer Pump, Tucson, AZ, USA). For enhancing the flow disturbution, a 0.23 cm-thick polypropylene spacer (Industrial Netting, Inc., Minneapolis, MN, USA) was assembled on each side. The feed and permeate pressures were about 20.7 kPa and 17.2 kPa, respectively. A heat mantle was used to heat the feed solution, and a cooler was used to cool the permeate. The water flux, J_w_, was measured by weight change of the permeate and recorded using a digital scale. Rejection, R_NaCl_, was determined by conductivity (Cond 3310, WTW, Oberbayern, Germany) and calculated using Equation (1) [25].

(1) $$R_{NaCl}\;\left(\%\right)=\left(1-\frac{\frac{V_{p}C_{p}}{J_{w}A_{m}t}}{C_{f}}\right)\times 100$$

where $V_{p}$ is the volume of permeate solution, $C_{p}$ is the salt concentration in the permeate, and$\;C_{f}$ is the salt concentration in the feed (3.5 wt% NaCl). $A_{m}$ is the active membrane area and t is time. Rejection values were determined over a 10 min time interval.

### 2.6. Antifouling and Antiwetting Investigation with Model Solutions

The antifouling test was performed using an oily saline solution. The 3.5 wt % NaCl solution was spiked with 1000 ppm crude oil (ONTA, Midland, TX, USA). To obtain a homogeneous oily saline solution, the solution was mixed at 16,000 rpm for 30 min using the homogenizer (PT1300D, Kinematica Inc., Bohemia, NY, USA). The oil droplet size in the resulting solution was ~5 µm in a range of 1.3–9 µm and the oil volume fraction was ~0.0014 vol%, with no change in droplet size over 48 h. Nevertheless, the oil suspension was prepared immediately before starting the experiment. Permeate weight was recorded every 10 min.

The antiwetting properties of the membrane were investigated by challenging the membrane with a surfactant solution. In these experiments, the MD system was operated with the feed stream consisting of a base 3.5 wt % NaCl solution. The system was run for 2 h. Next, SDS was added such that specified concentrations of 0.1, 0.2, and 0.4 mM were obtained in the feed solution. The system was operated at each SDS concentration for 1 h. The permeate and conductivity were monitored every 10 min.

Finally, the system was tested with a 3.5 wt% NaCl solution containing 1000 ppm crude oil and 100 ppm SDS. The concentration of SDS was lower than the critical micelle concentration (8.2 mM) [26]. Flux and conductivity were recorded every 10 min.

### 2.7. Membrane Performance with PW

PW water samples [3] were characterized at the Arkansas Water Resources Center, University of Arkansas. The results are given in Table 1. The PW was heated to 60 °C before starting the experiment. For each experimental run, the volume of feed solution was 500 mL. The experiment was stopped at 40% water recovery or if the conductivity of the permeate increased above 300 µs/cm. The conductivity and permeate flux were determined and recorded continuously by the computer.

## 3. Results and Discussion

### 3.1. Physicochemical Properties of the Nanofibrous Membranes

We have focused on casting electrospun membranes, given their unique features: re-entrant and tunable morphology, high porosity, pore interconnectivity, surface roughness, and hydrophobicity [25]. We have modified the surface chemistry of the membrane to minimize penetration of low surface tension solutions through the membrane and adsorption of low surface energy compounds [4]. In this study, the surface energy of the membrane was modified through FAS, as characterized by static contact angle analysis.

**Figure 3:**
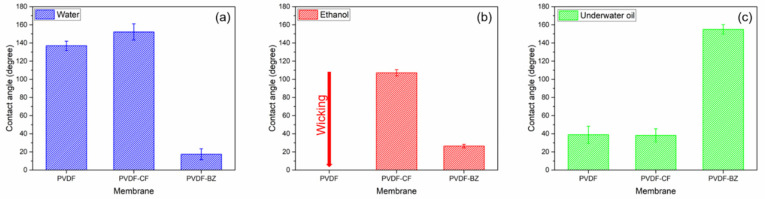
Water—(**a**), ethanol—(**b**), and oil–water (**c**) contact angles for PVDF, PVDF-CF and PVDF-BZ membranes. Droplet volume was 5 µL. The results represent the average of at least three measurements.

Figure 3 shows the surface contact angle results. Three solutions were used to characterize the membrane surface: DI water (surface tension (γ) = 72.8 mN/m), ethanol (γ = 22.4 mN/m), and mineral oil (γ ≈ 30 mN/m) [8]. The unmodified PVDF membrane is hydrophobic (~140°), and the air–water contact angle is as expected. The low energy fluorine surface is superhydrophobic (~150°), and the air–water contact angle is a result of the Cassie−Baxter state (low surface energy material and re-entrant fibrous structure) [17]. The low energy fluorine surface is confirmed by the ethanol–air contact angle (~110°) of the PVDF-CF membrane, where the ethanol droplet did not swell the membrane; on the contrary, the unmodified PVDF membrane showed fast wicking. For PVDF-BZ, a superhydrophilic surface was created, which was attributed to the strong charge density hydrophilic polymer pGS grafted on the surface.

In addition, to the in-air contact angles, the underwater oil contact angle was used to simulate actual operation when treating PW. The hydrophobic PVDF and PVDF-CF surfaces strongly attracted oil droplets due to hydrophobic–hydrophobic interactions. Here, we suppress the adsorption of oil by grafting pGS. Zwitterion coated surfaces have been used for forward osmosis [3,27], microfiltration [21,28], and ultrafiltration membranes [29]. However, their use to modify MD membranes is rare. The zwitterionic moiety showed excellent oil/water separation performance due to the presence of a hydrophilic surface. The sulfonic group-modified surfaces led to the formation of a strong hydration layer, which enhanced the surface antifouling and oleophobic properties [27,30]. From Figure 3c, it can be seen that the PVDF-BZ surface is superoleophobic with an underwater contact angle of around 155° [3]. Taken together, the contact angle measurements suggest that the PVDF-BZ membrane could be ideally suited for treating PW water by MD.

**Figure 4:**
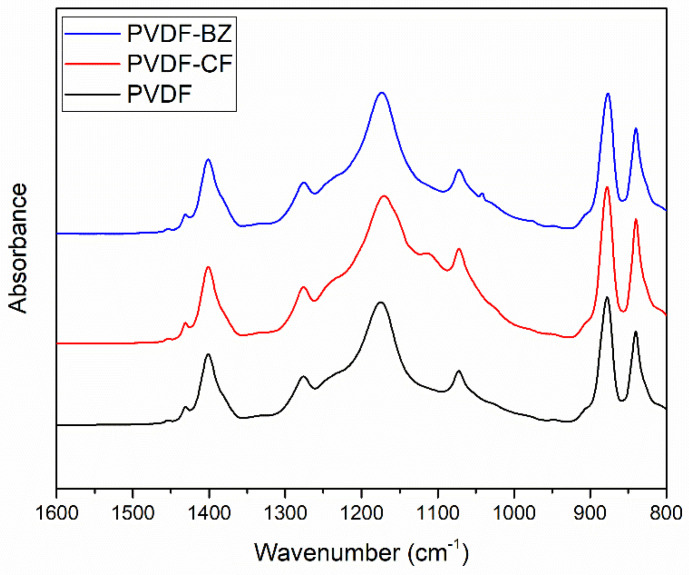
ATR-FTIR spectra of unmodified PVDF and modified membrane, PVDF-CF and PVDF-BZ.

The functional groups on the membrane surface were confirmed by ATR-FTIR. Figure 4 shows the FTIR spectra in a range of 1600–800 cm^−1^. The peaks around 1402 cm^−1^ and 1070 cm^−1^ on the PVDF membrane may be attributed to CH_2_ and C–C–C, respectively [29,31]. The new peak around 1110 cm^−1^ on the PVDF-CF membrane corresponds to silanol groups [4], confirming that silica nanoparticles were successfully grown on the fiber surface through electrostatic adsorption. The presence of the fluorine coating on the PVDF-CF membrane cannot be verified, as the peak at 1170 cm^−1^ represents CF_2_ on the PVDF-CF as well as the fluorine present in the base PVDF membrane. Although it was not evident that FAS fluorinated the surface of the silica particles, the antiwetting behavior based on the ethanol contact angle suggests fluorination of the surface of the silica particles (Figure 3). Finally, the small new peak on PVDF-BZ at around 1040 cm^−1^ may be attributed to the SO_3_^−^ of the zwitterionic polymer pGS. Thus, pGS grafting on to the surface of PVDF-BZ membrane is confirmed.

**Figure 5:**
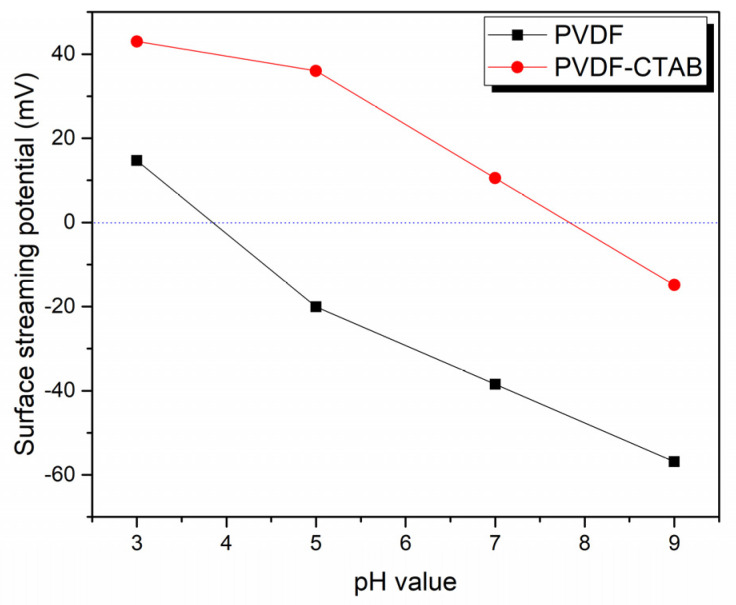
Surface streaming potential in different pH (3, 5, 7, 9) feed streams. PVDF represents the unmodified membrane and PVDF-CTAB indicates that blending CTAB in the PVDF doping solution leads to a more positive streaming.

Streaming potential analysis (Figure 5) over a pH range from 3 to 9 was used to evaluate membrane surface charge. The results indicate that after blending the positive surfactant, CTAB, into the casting solution, the membrane indeed exhibited a more positive surface than the PVDF membrane.

**Figure 6:**
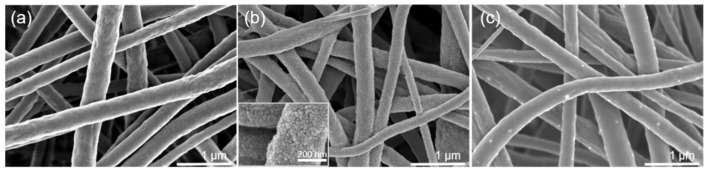
SEM images of (**a**) PVDF, (**b**) PVDF-CF, and (**c**) PVDF-BZ membranes. Scale bar is 1 µm.

Development of fouling resistant MD membranes for treating PW will require a surface morphology that provides a kinetic barrier to the adsorption of low surface energy compounds. Figure 6 is an SEM image of the PVDF, PVDF-CF, and PVDF-BZ membranes. The re-entrant fiber structure is clearly seen. The thickness of the PVDF, PVDF-CF, and PVDF-BZ membranes was 115 ± 10.8, 133.3 ± 12.5, and 138.3 ± 1 0.3 μm, respectively, as measured by a digital caliper (E-Base, Yunlin, Taiwan). The fiber diameter of the PVDF membrane is 393 ± 49 nm, which is larger than PVDF-CF (262 ± 31 nm). This is most likely due to the presence of CTAB in the doping solution, which increases the charge density of the solution. This in turn required the use of a higher voltage during the electrospinning process to overcome the solution surface tension, create a Taylor cone, and draw out the nanofiber, which will be thinner. The nanoparticles on the PVDF-CF membrane (Figure 6b) are clearly visible [4,32]. The size of a single nanoparticle is ~15 nm, matching the results obtained from DLS (Figure 2). Moreover, the superhydrophobic and omniphobic surface (Figure 3) was attributed to the re-entrant structure of the PVDF-CF membrane. The zwitterionic grafted nanofiber on the PVDF-BZ membrane surface displays some small dots, which represent the coated GS polymer. The size of the dots were between 13.5 nm and 27.3 nm, which is expected from our previous work for GS polymer coating [33]. These morphologies will change the surface and physicochemical properties and simultaneously alter the intrinsic membrane performance.

### 3.2. Intrinsic Membrane Performance

**Figure 7:**
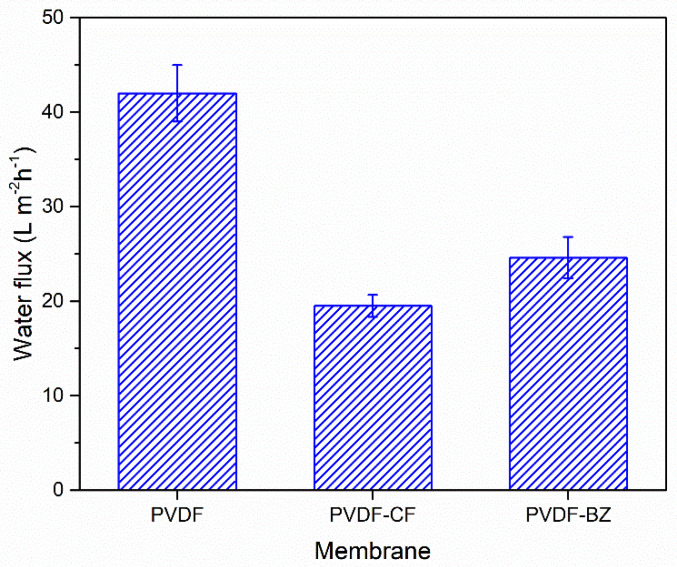
Permeate flux for PVDF, PVDF-CF, and PVDF-BZ membranes challenged with a feed stream consisting of a 3.5 wt% NaCl solution. The temperature of the feed and permeate streams was 60 °C and 20 °C, respectively. The error bars represent the range of triplicate results.

The permeate flux result is shown in Figure 7, for a feed stream consisting of a 3.5 wt% NaCl solution. The flux for the PVDF membrane is 42 ± 2.99 Lm^−2^h^−1^. As can be seen, there is higher flux for the PVDF-CF membrane (19.5 ± 1.16 Lm^−2^h^−1^) and the PVDF-BZ membrane (24.6 ± 2.19 Lm^−2^h^−1^). A unique feature of electrospun membranes is the presence of a highly interconnected porous mat structure. The highly interconnected porous channels provide a large number of paths for vapor transport, and hence high permeate fluxes [34]. In fact, all three membranes display permeate fluxes that are higher than most commercial flat sheet membranes. Literature data indicate permeate fluxes in the range of 5.1–10.6 Lm^−2^h^−1^ for most commercially available flat sheet membranes [35].

The PVDF-CF membrane has a lower flux than the PVDF and PVDF-BZ membranes due to the presence of smaller diameter fibers, leading to smaller vapor channels. In addition, the presence of FAS nanoparticles on the fiber surface further restricts the channel diameter. The zwitterion functionalized PVDF-BZ membrane displays a slightly increased flux compared to the PVDV-CF membrane. This is most likely due to the presence of a hydrophilic membrane surface that faces the feed solution. Importantly, salt rejection for all three membranes was above 99.9%. Thus, the presence of a thin hydrophilic membrane surface that faces the feed stream does not influence the salt rejection of the PVDF-BZ membrane.

### 3.3. Antiwetting and Antifouling Behavior

**Figure 8:**
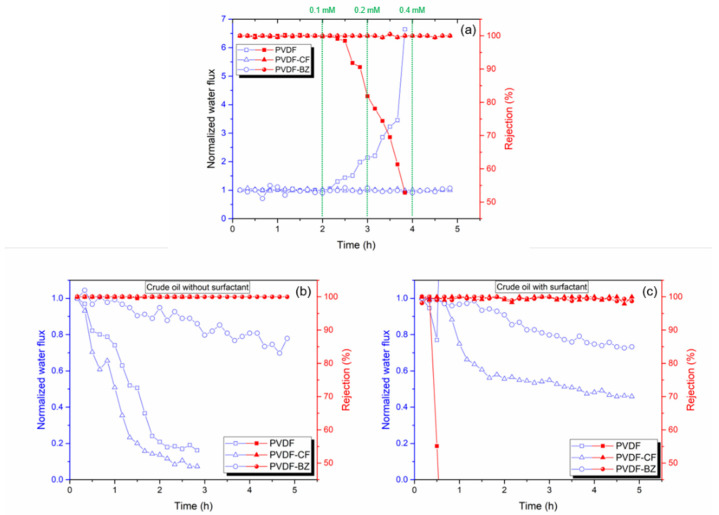
Effect of SDS and crude oil in the feed on membrane performance. (**a**) Antiwetting test of the MD membranes using a range of SDS concentrations. (**b**,**c**) Antifouling test using 1000 ppm crude oil suspension with/without surfactant solution. The feed and permeate flow rates were 0.86 and 0.35 mL/min, respectively.

Development of membranes that are resistant to wetting and fouling during MD to treat PW is a major challenge that has limited the commercial application of MD. Most previous studies improved MD membranes in two key aspects: antiwetting (by imparting omniphobicity) [4,16,36] and antifouling (by imparting hydrophilicity) [5,37]. Huang et al. [15]. state that a bilayer membrane could concurrently maintain good fouling and wetting resistance through spray modification. In this study, we also report a bilayer MD membrane. However, we have chosen for the first time to create a hydrophilic layer by grafting zwitterionic species through the epoxy ring-opening reaction. In order to test the antiwetting properties of the membrane, SDS is used as a model amphiphilic agent to induce membrane wetting. The results are shown in Figure 8a. The MD was run for 2 h using a 3.5 wt% NaCl feed solution containing increasing concentrations of SDS from 0.1 to 0.4 mM. In order to compare the change in flux during MD, the flux has been normalized by dividing by the initial flux over the first 10 min of operation. As expected, the PVDF membrane was rapidly wet by the SDS-containing feed solution. The flux for the PVDF membrane increased above 600% with 0.4 mM SDS present in the feed. The corresponding salt rejection decreased, indicating wetting of the membrane and direct passage of the feed through the membrane pores. The PVDF-CF and PVDF-BZ membranes, however, maintained 99% salt rejection during the entire test. In the case of the PVDF-BZ membrane, we note that the presence of a thin hydrophilic layer facing the feed stream did not lead to wetting.

To test the fouling resistance of the membrane, crude oil was added to the NaCl feed stream [37]. The PVDF and PVDF-CF membranes displayed rapid water flux decline within 3 h (Figure 8b), which was attributed to the hydrophobic attraction between the oil droplet and the membrane. The underwater oil water contact angles support this observation (Figure 3c). However, as the PVDF-BZ membrane contains a thin hydrophilic zwitterionic surface layer that contacts the feed stream, this suppresses the adsorption of oil. A significant improvement in flux was observed. The flux was above 70% of the normalized water flux during 5 h of operation. This is due to the superoleophobic surface that was created by the zwitterionic layer on the membrane surface that faces the feed solution [3,29,38]. It is important to note that all three membranes maintained a salt rejection over 99%.

Finally, membranes were tested with a 3.5 wt% NaCl solution containing 1000 ppm crude oil with 100 ppm SDS. The results are shown in Figure 8c. The unmodified PVDF membrane showed a rapid decrease in flux due to fouling by the oil, and then a rapid increase in flux and decrease in rejection due to wetting. The combined effect of the oil and SDS led to rapid membrane failure [37]. The PVDF-CF membrane resisted wetting. It maintained a salt rejection above 99% though adsorption of the oil, which led to fouling and a decrease in flux. Additionally, due to most of the oil being stabilized by SDS, the flux reduction was more moderate than in the absence of SDS. The same phenomena can explain the reduction in permeate flux for the PVDF-BZ membrane. However, the presence of a hydrophilic surface layer suppressed fouling, leading to a higher water flux than the PVDF-CF membrane. Consequently, PVDF-BZ is a promising MD membrane for treating high-saline oily wastewater as it showed an excellent salt rejection and antifouling properties simultaneously.

### 3.4. Treating PW

**Figure 9:**
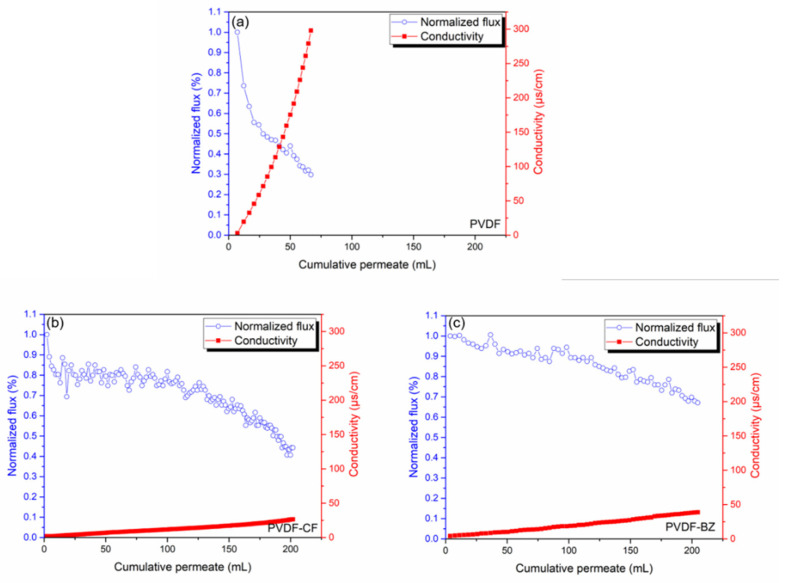
Flux and salt rejection for a produced water (PW) feed stream. (**a**) PVDF membrane, (**b**) PVDF-CF membrane, (**c**) PVDF-BZ membrane. The target water recovery was setup at 40%, or 200 mL permeate. The initial fluxes were (**a**) PVDF membrane: 38.88 Lm^−2^ h^−1^, (**b**) PVDF-CF membrane: 16.83 Lm^−2^ h^−1^, (**c**) PVDF-BZ membrane: 18.43 Lm^−2^ h^−1^.

Finally, the membranes were challenged with PW. Real PW is far more complex than the synthetic feed streams used to validate membrane performance. The TDS of the PW was 245,269 ppm, which is much higher than 3.5 wt% (35,000 ppm) NaCl used as the model feed solution. In addition, the PW contains oil and grease, forming agents, surfactants, etc. which are far more challenging to membrane stability. The results of the PW analysis are given in Table 1. The normalized permeate flux (normalized by dividing by the initial flux over the first 10 min of operation) for the PVDF membrane (Figure 9a) declined rapidly to 0.3 before 70 mL of permeate had been collected, indicating rapid fouling of the membrane. In addition, the conductivity of the permeate increased rapidly, indicating pore wetting and passage of the feed directly to the permeate. These observations are in keeping with the results of the model feed streams. However, the degree of fouling is much greater due to the presence of a large number of organic foulants in the PW. The results indicate that the model feed streams developed here may be used when optimizing membrane properties [2,4,5,8].

In contrast, results for the PVDF-CF and PVDF-BZ membranes indicate a much slower increase in permeate conductivity, which may be attributed to the low surface energy and hierarchical re-entrant structure of the substrate, which suppresses wetting. Importantly, the decrease in permeate flux is much less for the PVDF-BZ membrane, indicating a much stronger resistance to fouling by the hydrophilic surface that faces the feed stream. Consequently, the productivity (amount of PW treated) by the PVDF-BZ membrane was greater the PVDF-CF membrane.

Our results indicate that the development of bilayer electrospun membranes where the surface properties of the membrane surface facing the feed and permeate sides are tuned to minimize fouling and wetting will improve membrane performance. The practical viability of these membranes will depend on maximizing the productivity of the membrane. However, the commercial viability of MD for treating PW depends on other factors such as the operation cost of the process versus current PW disposal methods such as trucking and deep well injection.

The integration of MD into a multi-unit operation PW treatment process will be required. Pre-treatment of the feed prior to MD will be likely, leading to the development of a hybrid process containing MD. Thus, it will be important to study the overall process [39]. For example, in recent years, processes such as FO-MD, combining the advantages of these two-unit operations (FO: high fouling resistance; MD: high salt rejection), has been proposed. In addition, electrocoagulation-MD [3] where electrocoagulation is used to reduce foulants in the feed stream before MD has been investigated [9]. MD integrated with crystallization for treating PW has been proposed as a way to maximize water recovery and control inorganic scaling [40]. Further, it will be necessary to develop appropriate operating strategies to ensure the onset of scaling is detected and membrane regeneration commences. If scale formation progresses too far, the membrane will fail, and regeneration will not be feasible. Thus, while many studies have focused on the development of membranes tailored for MD when challenged with PW, the development of a practical technology to treat PW remains challenging.

## 4. Conclusions

The novel bilayer MD membrane developed here, PVDF-BZ, consisted of PVDF-CTAB and PVDF nanofibers. The surface properties of PVDF-CTAB nanofibers that face the permeate stream were modified to create an omniphobic surface. The surface properties of the PVDF nanofibers that face the feed stream were modified to create a hydrophilic surface. The omniphobic surface contained silica nanoparticles that were salinized to lower their surface energy. The hydrophilic surface contained a zwitterionic polymer, poly(glycidyl methacrylate-sulfobetaine methacrylate), which was grafted to the alkali-treated PVDF nanofiber through the “grafting to” modification, which is reported for the first time in this publication. The resulting omniphobic membrane surface displays an underwater oil contact angle of >150°. The membranes were challenged with PW obtained from hydraulic fracturing operations. Very good fouling and wetting resistance was observed for synthetic solutions. For actual PW, a decrease in the rate of fouling was observed over a 4-h period. These results indicate the importance of testing membranes with actual PW under industrially relevant conditions. Our results indicate the importance of tuning the membrane surface properties of the surfaces that face the feed and permeate streams in order to maximize flux, salt rejection and productivity. Electrospun membranes are attractive for MD as they contain a re-entrant structure which provides a kinetic barrier to the adsorption of low surface energy compounds such as surfactants.

## Figures and Tables

**Table 1:** Produced water (PW) analysis indicating main components.

Parameter	Unit	Value
Total dissolved solids (TDS)	mg L^−1^	245,000
Total organic carbon (TOC)	mg L^−1^	120
Total suspended solids (TSS)	mg L^−1^	131
Turbidity	NTU’s	6.0
pH	-	6.7
Chloride	mg L^−1^	147,000
Sulfate	mg L^−1^	478
Boron	mg L^−1^	97.4
Calcium	mg L^−1^	30,500
Magnesium	mg L^−1^	5450
Potassium	mg L^−1^	4330
Sodium	mg L^−1^	55,900
Conductivity	µS/cm	323,000
Total nitrogen	mg L^−1^	43.5
